# Lower limb amputations in Trondheim, Norway: A 40% reduction in diabetic major lower-limb amputations from 1996 to 2006

**DOI:** 10.3109/17453674.2010.519164

**Published:** 2010-11-26

**Authors:** Eivind Witsø, Arne Lium, Stian Lydersen

**Affiliations:** ^1^Department of Orthopaedic Surgery, St. Olav's University Hospital; ^2^Norwegian University of Science and Technology, Trondheim, Norway

## Abstract

**Background and purpose:**

In the city of Trondheim, Norway, diabetic lower-limb amputations accounted for one-third of all lower-limb amputations (LLAs). In an attempt to reduce this rate, a diabetic foot team was established in 1996. We present the incidence of LLA in Trondheim as measured 10 years later.

**Patients and methods:**

In 2004–07, we registered all LLAs performed in Trondheim and then compared the data with previously published data from 1994–1997. From 1996 through 2006, we registered the activity of the diabetic foot team and we also registered the number of vascular procedures performed on citizens of Trondheim from 1998 through 2006.

**Results:**

Comparing the two 3-year periods 1994–97 and 2004–07, we observed a decrease in all non-traumatic LLAs. The incidence of diabetic major LLAs per 10^3^ diabetics per year decreased from 4.0 to 2.4, and in patients with peripheral vascular disease we observed a decrease in LLAs from 18 to 12 per 10^5^ inhabitants per year. 5,915 consultations on diabetic subjects were conducted by the diabetic foot team during the period 1996–2006. From 1998 to 2006, the rate of vascular procedures decreased in the non-diabetic population, and was unchanged in diabetic subjects.

**Interpretation:**

In the population of Trondheim city there appears to have been a reduction in the rate of vascular obstructive lower-limb disease between the two 3-year periods 1994–97 and 2004–07. In our judgment, the decline in diabetic LLA also reflects better care of the diabetic foot.

During the last decade, several studies have been published on the decreasing incidence of lower limb amputations (LLAs) in the diabetic population (Holstein et al. 2000, Calle-Pascual et al. 2001, van Houtum et al. 2004, Ekelinen et al. 2006, Trautner et al. 2007, Larsson et al. 2008, Canavan et al. 2008). Although the rate of LLA due to peripheral vascular disease in the non-diabetic population has shown a similar trend (Holstein et al. 2000, Calle-Pascual et al. 2001, Ekelinen et al. 2006), unchanged or even increased rates of non-diabetic LLA have been reported (Trautner et al. 2007, Canavan et al. 2008). The reduction in the number of amputations in diabetics has been attributed to better preventive care for the diabetic foot in general, and in particular to the establishment of multidisciplinary diabetic foot teams and implementation of the guidelines of the International Working Group on the Diabetic Foot (1999). In addition, most reports also attribute the falling rate of LLAs in diabetics to an increasing number of vascular interventions. However, only a few authors have actually registered the rate of revascularization in the diabetic and non-diabetic population (McCaslin et al. 2007). In the 3-year period 1994–1996, the total number of primary LLAs in the city of Trondheim was 143. The incidence of primary LLA was 33 per 10^5^ inhabitants per year, and diabetic amputations accounted for one-third of all primary LLAs (Witsø and Rønningen 2001). Partly as a consequence of this registration, a multidisciplinary diabetic foot team was established at St. Olav's University Hospital in Trondheim in January 1996. The team has been an integrated part of the outpatient clinic of the Department of Orthopedic Surgery, and the main activity of the team is prevention and early treatment of diabetic foot ulcers.

Here we report on the prospective registration of all primary LLAs and re-amputations performed on patients who were citizens of Trondheim in the period 2004–2007. Data from this registration were compared with data from the amputation registration in the city of Trondheim that was performed 10 years earlier. We also registered the activity of the diabetic foot team during the decade 1996–2006, and the number of invasive and noninvasive vascular procedures of the lower extremity performed in diabetic and non-diabetic subjects during the same period.

## Patients and methods

We have previously published data on LLA in Trondheim for the period 1994–1997 (Witsø and Rønningen 2001). From April 1, 2004 through April 1, 2007 we registered all lower-limb amputations (LLAs) performed at St. Olav's University Hospital. This is the only hospital in Trondheim, and all amputations are performed at the Department of Orthopedic Surgery. As in 1994–97, we consecutively registered all primary amputations and re-amputations (see definitions). Auto-amputations were not included in the study. The registrations were based on 3 information sources: (1) consecutive registration of all LLAs, (2) operating theater records, including the operating theater at the outpatient clinic, and (3) hospital discharge data.

The appropriate level for amputation was determined by clinical evaluation. Patients without a diagnosis of diabetes mellitus (DM) but with foot ulcer and peripheral neuropathy were screened by measurement of blood sugar and HbA1_c_.

### Demographic data

During the 2 study periods, 1994–97 and 2004–07, the average number of inhabitants in Trondheim was 143,300 and 159,000, respectively. In both study periods, women constituted 51% of the population. In 1995 and 2005, respectively, the proportions of the total population that were in the following age groups were: 60–69 years, 8.0% and 7.8%; 70–79 years, 7.1% and 5.8%; 80–89 years: 2.8% and 3.4%; and ≥ 90 years, 0.4% and 0.5%, respectively.

The assessment of the diabetic population in Trondheim in 1994–97 was based on the the Nord-Trøndelag Health Study 1995–97 (HUNT 2). The 2 main counties of the middle-part of Norway are Sør- and Nord-Trøndelag. Trondheim is the largest city in Sør-Trøndelag. The Nord-Trøndelag Health Study (HUNT) is one of the largest health studies ever performed (www.ntnu.no/hunt/english). HUNT 2 was the second health survey in Nord-Trøndelag, and the third (HUNT 3) was completed in June 2008. In the HUNT 2 study, 71% of the Nord-Trøndelag population aged ≥ 20 years participated (Holmen et al. 2003). The highest degree of participation was in the female group aged 60–69 years (87%), and the lowest was in the male group aged 20–29 years (43%). The prevalence of DM was presented in the age groups 13–19, 20–29, 30–39, and so on up to 90+ years for men and women (Midthjell et al. 1999). In addition, a 0.15% prevalence of DM in children of < 13 years was based on the Norwegian Diabetes Register (Joner 1994). Data from Statistics Norway (www.ssb.no/english/) allowed us to make age groups (0–12, 13–19, 20–29, and so on up to 90+ years) for females and males in Trondheim in 1995. We applied the age-specific prevalence of DM in men and women ≥ 13 years in Nord-Trøndelag to the population of Trondheim that was ≥ 13 years, and a 0.15% prevalence of DM in individuals aged < 13 years in Trondheim. This resulted in an estimate of 2,870 inhabitants with DM in Trondheim in 1995.

Stene et al. (2004) evaluated the changing prevalence of DM in Norway. In that paper the figures from 6 different Norwegian studies in the period 1984–2001 were used to estimate the annual increase in the prevalence of DM as 1.4% in people aged ≥ 30 years. From the same studies, it was possible to estimate a sex-specific increase in the prevalence of diabetes of 0.4% for women and 2.5% for men. Thus, the prevalence of DM in Trondheim in 2004–2007 was estimated as follows. Briefly, in females in the 0- to 19-year age group, the prevalence of DM in 1994–97 was 0.24%, which we considered to be unchanged in 2004–07. In females ≥ 20 years of age, the prevalence in 1994–97 was 2.8%, which by 2004–07 had increased to 2.9%. In males in the 0- to 19-year age group, the prevalence of DM in 1994–97 was 0.30%, which we considered to be unchanged in 2004–07. In males ≥ 20 years, the prevalence in 1994–97 was 2.4%, which had increased to 3.1% by 2004–07. These figures allow an estimate of 3,610 subjects with DM in Trondheim in 2004–07.

### Activity of the diabetic foot team

The diabetic foot team of Trondheim was first and foremost a medical service for the inhabitants of Sør-Trøndelag County, most of whom are citizens of Trondheim. When the foot team was established in 1996, this team in collaboration with the Departments of Endocrinology, Vascular Surgery, and Dermatology, agreed on a policy whereby all patients with a diabetic foot ulcer would be referred to the foot team or directly to the Department of Orthopedic Surgery. Information about the foot team was sent to healthcare workers in the county, in particular to general practitioners and community nurses. Finally, meetings were arranged to inform people about the activity and policy of the team. The members of the team were nurses, podiatrists, orthopedic surgeons, orthotists, prosthetists, and plaster technicians. At the first consultation, each patient received an information sheet for tuition and instruction. Screening for neuropathy was performed with a 10-gram Semmes-Weinstein monofilament and a 128 Hz tuning fork. Patients with a foot ulcer and no palpable pulses at the ankle level were immediately referred to the Department of Vascular Surgery. In addition to information and preventive care, the main treatment strategy was off-loading of neuropathic ulcers, vascular intervention in cases of neuroischemic ulcers, and radical debridement in cases of chronic osteomyelitis.

### Vascular interventions

The Norwegian vascular registry (NorKar) was established in 1995. Most departments performing vascular surgery in Norway report to this registry, and the completeness of data is at least 85% (Dahl et al. 2006). Based on the local database of NorKar at St. Olav's University Hospital, we identified the annual number of femoropopliteal and femorodistal bypasses performed at St. Olav's during the period 1998–2006.

From 1998, all percutaneous vascular procedures performed at the Department of Radiology, St. Olav's University Hospital, were registered in a protocol. We identified all patients on which percutaneous transluminal angioplasties (PTAs) had been performed on arteries distally to a. iliaca communis during the period 1998–2006. Only patients who were citizens of Trondheim were included.

### Definitions

When we planned the present study, we decided that the overall design should be as similar as possible to that of the study from 1994–1997. In that study, the definitions of *primary amputation* and *re-amputation* were based on a paper by Larson and Apelqvist (1995).

*Primary amputation:* the first amputation procedure in a sequence until a final outcome (healed or death). Consequently, primary amputation is included the following: initial amputation, first amputation, first-event amputation, new amputation, and second leg amputation—i.e. all amputations except re-amputations.

*Re-amputation:* amputation of a limb with an unhealed previous amputation by cutting through bone or a joint.

*Major amputation:* amputation through or above the ankle joint.

*Minor amputation:* Amputation below the ankle joint.

*A diabetic subject:* a person treated for high blood sugar using diet, oral antidiabetics, or insulin.

*Diabetes mellitus types I and II:* patients were classified as having DM type I if diabetes was diagnosed at the age of < 40 years *and* if insulin treatment was started less than 6 months after diagnosis.

*A diabetic foot ulcer:* infection, ulceration, destruction of deep tissue of the foot associated with neuropathy and/or peripheral arterial disease of the lower extremity of people with DM.

### Statistics

If not otherwise stated, the results are presented as arithmetic mean with dispersion (as total range) in parentheses. Incidence rates were analyzed using Poisson regression with decade as covariate, using Stata software version 10.0. Estimates are given with 95% confidence intervals (CIs) where relevant. Wilson (score) confidence intervals for proportions were computed. Two-sided p-values of < 0.05 were considered significant.

## Results

During the 2 separate study periods (1994–1997 and 2004–2007), 33 and 24 primary LLAs/10^5^ inhabitants/year were performed in Trondheim (p = 0.007). The mean ages of the amputees were 73 (21–98) years in 1994–97 and 74 (33–93) years in 2004–07. Of the amputees, 67/143 (47%, CI: 39–55) were females in 1994–97 and 49/113 (43%, CI: 35–53) were females in 2004–07.

We identified 3 diagnostic groups: (a) peripheral vascular disease (PVD) amputations, (b) diabetic amputations, and (c) other LLAs (this group included cases of trauma, invasive soft tissue infections, cold injury, and malignant tumors, amongst others). Diabetic LLAs accounted for one-third of the amputations in both periods. We observed a 40% reduction in the annual rate of major diabetic LLAs/10^3^ diabetics. However, we observed a similar reduction in the rate of PVD LLAs (Table 1). Consequently, in both periods the incidence of primary LLA in diabetic subjects was approximately 30 times higher than the incidence of primary LLA in non-diabetic subjects. In both periods, patients with DM were amputated at a younger age compared to patients with PVD: 71 (42–97) years vs. 78 (58–98) years in 1994–97, and 73 (34–93) years vs. 79 (47–93) years in 2004–2007 (p = 0.001 and p = 0.03, respectively; Student's t-test).

**Table 1.** Primary LLAs performed on citizens of Trondheim in 1994–1997 and 2004–2007

	1994-01-01 to 1997-01-01	2004-04-01 to 2007-04-01	p-value for difference
Inhabitants	143,000	159,000	
Inhabitants with diabetes mellitus	2,870	3,610	
All primary LLAs	143	113	
Diabetic primary LLAs	47	38	
Primary LLAs from peripheral vascular disease (PVD)	76	55	
Other primary LLAs	20	20	
Primary LLAs/10^5^ inhabitants/year	33	24	0.007
Diabetic primary LLAs/10^5^ inhabitants/year	11	8	
Diabetic primary LLAs/10^3^ diabetics/year	5.5	3.5	0.04
Major diabetic primary LLAs/10^3^ diabetics/year	4.0	2.4	0.06
Primary LLAs from PVD/10^5^/year	18	12	0.02

The relative rate of major LLA/all LLA was similar in the two study periods: 118/143 (83%, CI: 75–88) in 1994–97 and 92/113 (81%, CI: 73–88) in 2004–07. However, the incidence of primary transtibial amputations in patients with DM decreased from 3.7/10^5^ inhabitants/year in 1994–97 to 1.7/10^5^ inhabitants/year in 2004–07. The incidence of primary transfemoral amputation in patients with PVD decreased from 6.8/10^5^ inhabitants/year in 1994–97 to 1.7/10^5^ inhabitants/year in 2004–07. In both periods, major LLAs were more common in patients with PVD than in patients with DM (Table 2).

**Table 2.** All primary LLAs performed on citizens of Trondheim in 1994–97 and 2004–07 due to diabetes mellitus (DM), peripheral vascular disease (PVD), or for other reasons (such as trauma, infection, or malignant tumors)

	LLA due to DM		LLA due to PVD		LLA for other reasons	
	1994–97	2004–07	1994–97	2004–07	1994–97	2004–07
Partial foot amputation	13	12	3	3	9	6
Ankle disarticulation	0	0	0	0	1	1
Transtibial amputation	16	8	12	17	3	5
Knee disarticulation	14	12	31	27	3	1
Transfemoral amputation	4	6	29	8	3	5
Hip disarticulation	0	0	1	0	1	2
Major LLA	34	26	73	52		
All LLA	47	38	76	55		
Ratio	0.7	0.7	1.0	1.0		
95% CI	0.6–0.8	0.5–0.8	0.9–1.0	0.9–1.0		

More than 85% of all major LLAs in both periods were performed at age ≥ 70 years in females and ≥ 60 years in males (Table 3). The rate of re-amputation was 22/143 (15%, CI: 10–22) in 1994–97 and 21/113 (19%, CI: 12–27) in 2004–07. The rates of re-amputation at the partial foot, transtibial, and knee disarticulation amputation levels in patients with DM and PVD are presented in Table 4.

**Table 3.** Breakdown of major LLA in Trondheim in 1994–97 and 2004–07 in terms of patient age and sex

Age		< 60 years	60–69 years	≥ 70 years
1994–97	Male	5	16	41
	Female	3	4	49
2004–07	Male	8	7	37
	Female	1	2	37

**Table 4.** Re-amputations at the partial foot, transtibial, and knee disarticulation level in patients with diabetes mellitus (DM) and peripheral vascular disease (PVD)

Level	1994–1997 Re-amputations / total (rate, CI)	2004–2007 Re-amputations / total (rate, CI)
Partial foot amputation		
Patients with DM	5/13 (0.4, CI: 0.2–0.6)	3/12 (0.3, CI: 0.1–0.5)
Patients with PVD	2/3 (0.7, CI: 0.2–0.9)	1/3 (0.3, CI: 0.1–0.8)
Transtibial amputation		
Patients with DM	3/16 (0.2, CI: 0.1–0.4)	3/8 (0.4, CI: 0.1–0.7)
Patients with PVD	2/12 (0.2, CI: 0.0–0.4)	3/17 (0.2, CI: 0.1–0.4)
Knee disarticulation		
Patients with DM	1/14 (0.1, CI: 0.0–0.3)	4/12 (0.3, CI: 0.1–0.6)
Patients with PVD	5/31 (0.2, CI: 0.1–0.3)	5/27 (0.2, CI: 0.1–0.4)

779 diabetic subjects were referred to the diabetic foot team in Trondheim over the period 1996–2007. 5,915 consultations were held and, except for the first year, the activity of the team was stable with mean 600 (502–666) consultations per year. 5/779 patients (1%) had DM secondary to pancreatic trauma, 182/779 patients (23%) had DM of type I and 592/779 (76%) had DM of type II. In patients with type-I and type-II DM, peripheral neuropathy was registered in 115/182 (63%, CI: 56–70) and 514/592 (87%, CI: 84–89) patients. At the first consultation, 41/182 (23%, CI: 17–29) patients with type-I DM had a diabetic foot ulcer, as compared to 223/592 (38%, CI: 34–42) patients with type-II DM. Of the 779 patients who were referred to the diabetic foot team, approximately 460 were citizens of Trondheim, and 160 (35%) of these patients were diagnosed as having either a neuropathic or a neuroischemic foot ulcer at the first consultation.

During the period 1998–2006, the rate of femoropopliteal and femorodistal bypasses and the rate of PTA performed on arteries distal to a. iliaca communis decreased in patients with PVD. The rate of vascular procedures was unchanged in diabetic subjects (Table 5).

**Table 5.** Annual numbers of invasive and noninvasive vascular procedures performed in diabetic and non-diabetic subjects in Trondheim, 1998–2006

A	B	C	D	E	F	G
1998	3,074	142,704	8	18	9	40
1999	3,146	144,041	8	14	8	46
2000	3,219	145,640	9	11	10	48
2001	3,293	146,873	7	9	4	42
2002	3,370	148,038	9	15	10	47
2003	3,448	149,251	7	11	15	39
2004	3,528	150,823	8	10	12	30
2005	3,610	152,551	9	7	13	30
2006	3,694	154,919	9	6	12	20
Relative change in incidence per year **^a^**			–0.013	–0.107	0.046	–0.080
Estimate (upper 95% CL)			–0.096	–0.174	–0.034	–0.117
Estimate (lower 95% CL)			0.078	–0.036	0.133	–0.041
p-value			0.8	0.004	0.3	< 0.001

**^a^** for example, –0.013 means a relative reduction of 1.3% per year. Computed as exp(beta) – 1, where beta is the coefficient for year in Poisson regression with the relevant population as exposure variable. A Year B Diabetic population, estimates based on 2,870 and 3,610 diabetics in 1995 and 2005, assuming a constant growth rate. C Non-diabetic population, total population from Statistics Norway (www.ssb.no/english/) minus the estimated diabetic population in the preceding column D Femoropopliteal and femorodistal bypasses in diabetic subjects E Femoropopliteal and femorodistal bypasses non-diabetic subjects F PTA in diabetic subjects G PTA in non-diabetic subjects

## Discussion

During the decade 1994–97 to 2004–07, the incidence of primary LLA declined in the city of Trondheim in both main diagnostic groups, i.e. patients with diabetes mellitus and patients with peripheral vascular disease.

The completeness of the patient material is important in a registry (Larsson et al. 2008). Studies on the incidence of LLA have mainly been retrospective and based on hospital registers; not all studies have included all amputation levels and some studies have not distinguished between first amputation, primary amputation, and re-amputation, and, finally, not all studies have had a clear definition of DM (Lázlo et al. 1999, Mayfield et al. 2000, Calle-Pascual et al. 2001, Eskelinen et al. 2001, Trautner et al. 2001, Romagnoli et al. 2003, Ekelinen et al. 2004, Carmona et al. 2005, Harding 2005, Ekelinen et al. 2006, McCaslin et al. 2007). The uncertainty of the prevalence of DM in the study population is a particular problem related to registration of diabetic LLA (van Houtum and Lavery 1997).

In the present study and in the study from 1994–1997 (Witsø and Rønningen 2001), similar methods of patient registration were employed. In theory, inhabitants of Trondheim could have been amputated at other hospitals outside Trondheim. During the last 20 years, we have been aware of a few cases of trauma and malignant tumors (osteosarcoma) when inhabitants of Trondheim were amputated at hospitals outside Trondheim. However, in cases of amputation due to DM and PVD, this should be extremely rare.

There is wide variation in how data from studies on the incidence of LLA in general, and on diabetic LLA in particular, are presented. In incidence reports, the denominator might either be the total population or the number of diabetic subjects. The numerator is presented as the total number of amputations (including re-amputations), or only the first or primary amputation. Major amputation may be defined as amputations proximal to the tarsometatarsal joint, proximal to the subtalar joint, or proximal to the ankle joint (Larsson et al. 1995, Holstein 2000, Calla Pasqual 2001, Eskelin 2006, Canavan 2008, Larsson 2008). In both study periods we registered primary LLA, and primary LLA (either all or major) was employed as the numerator in incidence estimates. In primary LLA, we included all amputations except re-amputations. Primary amputation was chosen as a variable since this allowed us to compare amputation rates in the 2 study periods. Hence, data from the present study cannot readily be compared with other amputation studies on first or initial LLA, and studies where all amputations also include re-amputation (The Global Lower Extremity Amputation Study Group 2000, Calle-Pascual et al. 2001, Canavan 2008, Johannesson et al. 2009). However, when the data from our study were compared with those from other relevant studies, we found that the incidence of all LLAs and diabetic amputations had fallen from intermediate rates in 1997–1997 to low rates in 2004–2007 (Jeffcoate and van Houtum 2004).

Contrary to other studies, the falling incidence of diabetic and peripheral vascular disease LLA in Trondheim cannot be attributed to an increased number of vascular interventions (Larson et al. 1995, Holstein et al. 2000, Eskelinen 2004, Eskelinen et al. 2006, Larsson et al. 2008). In non-diabetic inhabitants of Trondheim, there was even a decline in the rate of invasive and non-invasive vascular procedures in the lower extremity during the period 1998–2006. The annual change in incidence shown in Table 5 could have been influenzed by changes in sex or age distribution. However, the sex distribution in the 2 study periods was stable at 51% females and 49% males, and there were only slight changes in age distribution, as described under Demographic data. The proportion of patients aged 80 years and above increased from 3.3% to 3.9%, and the proportion aged 60–79 decreased from 15.1% to 13.6%. The relative change per year in incidence rates in patients with PVD (Table 5), correspond to a reduction of the rate of femoropopliteal and femorodistal bypasses and PTA of approximately 50% from 1998 to 2006. Thus, we consider the possible influence of change in age distribution to be negligible.

The results of 2 health survey studies from the middle-part of Norway showed a strong decline in mortality from coronary heart disease between the periods 1984–86 and 1995–97 (Dale et al. 2008). Although the decline was observed in people with and without diabetes, the 2-fold higher mortality rate from coronary heart disease in diabetics did not change over time. During the past 3 decades, a reduced incidence of coronary heart disease has been observed in other Nordic countries (Abildstrom et al. 2003, Laatikainen et al 2005). This decrease has been attributed to reduced exposure to risk factors such as elevated serum cholesterol, smoking, and elevated blood pressure. In contrast, an unfavorable trend in the incidence of coronary heart disease in young adults in the USA has been explained by considerable increases in abdominal obesity, diabetes, and hypertension (Ford and Capewell 2007). The observed decrease in all non-traumatic LLA in our study may therefore have been a result of improvement in lifestyle in general and a reduced prevalence of vascular obstructive disease. The atherosclerotic lesions are located more distally in the lower extremity in diabetic subjects than in non-diabetic subjects (International Working Group on the Diabetic Foot 1999). Consequently, a reduced prevalence of atherosclerosis in the diabetic and non-diabetic population is in accordance with the 54% reduction in transtibial amputations seen in diabetic subjects, and the 75% reduction in transfemoral amputations in non-diabetic subjects. We speculated whether a more restrictive policy regarding LLA in the very elderly could explain the observed decline in LLA. However, the mean age of PVD amputees was similar in the 2 study periods (78 years and 79 years).

A diabetic foot ulcer is the major risk factor for LLA in diabetic subjects (Margolis et al. 2005). Prevention and early treatment of diabetic foot ulcers can reduce the amputation rate by 50%, and the establishment of a multidisciplinary foot care team is a cornerstone in the management and prevention of diabetic foot ulcers (Apelqvist et al. 2008). In a study from Germany lasting over 15 years (1990–2005), the estimated reduction in diabetic LLA above toe level was 37%, while the incidence rates in the non-diabetic population did not change. The observed decline in diabetic LLA was explained by better care in patients with diabetic foot pathology (Trautner et al. 2007). Although diabetic subjects are influenced by the same positive predictors as non-diabetic subjects, we partly attribute the decline in diabetic amputations in Trondheim to the activity of the diabetic foot team. In accordance with previous reports, we have observed that patients with DM of type II are particularly at risk (Morris et al. 1998). Patients with DM of type II have an increased risk of diabetic foot ulcers than patients with DM of type I. To avoid ulcers in these patients, collaboration with primary healthcare in general and general practitioners in particular, is important.

In the period 2004–2007, the rate of re-amputations was rather high in transtibial and knee disarticulation amputees. Although we did not observe a statistically significant increase compared to the period 1994–1997, a 0.3 rate of re-amputations after knee disarticulation in patients with DM is high. A similar regime for antibiotic prophylaxis was employed during both study periods. In 2004–2007, the amputation level was mainly determined by clinical evaluation. The reported re-amputation rate highlights the need for additional, noninvasive methods for assessment of the amputation level. In most cases of re-amputation after knee disarticulation we observed impaired circulation in the anterior flap, underscoring the need for a well-circulated posterior flap. Compared to other studies on LLA in Scandinavia, knee disarticulation is a more common amputation level in Trondheim (Larsson et al. 2008, Johannesson et al. 2009). At our clinic, we have a tradition of performing knee disarticulation (if possible) instead of transfemoral amputation. Such a strategy could of course result in too many re-amputations at the knee level, particularly if noninvasive methods for the assessment of arterial circulation are employed.

The present study has some limitations. First, the numbers of diabetics were not known accurately, and have been estimated. We have carefully explained the background to these estimates, and we consider them to be precise enough for our calculations. Secondly, due to a probable decrease in atherosclerosis and obstructive vascular diseases in the study population, we should have been able to observe a relative decline in the rate of diabetic LLA preceded by neuroischemic foot ulcers. The 1994–1997 study was not designed for such an analysis. Finally, we are unable to estimate the relative influence of the activity of the diabetic foot team and the general improvement in public health on the reduction in diabetic LLA.

In conclusion, in the city of Trondheim the rates of both diabetic- and PVD-associated primary LLA have decreased over the last decade. As in other studies, our results may reflect an improved quality of prevention and treatment of diabetic foot ulcers. In contrast to other studies, however, our results cannot be attributed to an increased rate of vascular intervention. To our knowledge, our study is one of the first reports of a reduced incidence of all non-traumatic LLA—probably due to a general improvement in public health in general and a reduced incidence of vascular obstructive disease in particular. Since diabetic subjects are still at 30 times higher risk of being amputated compared to non-diabetic subjects, a further reduction in the rate of diabetic LLA should be possible.
